# The serum galectin-3 levels are associated with the severity and prognosis of ischemic stroke

**DOI:** 10.18632/aging.202610

**Published:** 2021-03-03

**Authors:** Jia-Jun Zhuang, Li Zhou, Yan-Hua Zheng, Yan-Sheng Ding

**Affiliations:** 1 Department of Neurology, Weifang People’s Hospital, Weifang, Shandong, China; 2 Department of Clinical Laboratory, Weifang People’s Hospital, Weifang, Shandong, China

**Keywords:** galectin-3, acute ischemic stroke, prognosis, Chinese

## Abstract

Galectin-3, a microglia/macrophage-derived inflammatory mediator, plays a role in the stroke progression. In this single-center prospective study, we included 288 consecutive patients with a first-ever acute ischemic stroke to assess the association between galectin-3 serum level and clinical severity at admission and outcome at discharge by univariate and multivariate logistic regression. The results were presented as odds ratios (OR) and 95% confidence intervals (CI). Patients with high severity and poor outcomes had higher serum levels of galectin-3 (P<0.001 and P<0.001). Multivariate analysis suggested that a galectin-3 serum level in the highest quartile (The lowest three quartiles[Q1-3] as the reference) was associated with poor functional outcome (OR, 3.15; 95% CI, 2.44–3.87). The AUC (standard error) for the NIHSS and the combined model were 0.764 (0.031) and 0.823 (0.027), corresponding to a difference of 0.059 (0.004). This study shows that higher serum levels of galectin-3 are associated with stroke severity at admission and stroke prognosis at discharge in ischemic stroke.

## INTRODUCTION

Galectins are an ancient lectin family, and galectin-3 is a structurally unique member [1]. Galectin-3 is involved in many pathophysiological conditions [1], including metastasis [2], inflammation reactions [3], immunity [4], and cancer [5]. The role of Galectin-3 in the development and progression of heart failure [6] has been reported. Another study found that galectin-3 could be used as a biomarker to assess heart failure patients [7]. Furthermore, one study showed that as a macrophage-derived mediator, galectin-3 could induce cardiac fibroblast proliferation and ventricular dysfunction [8].

Stroke is one of the main causes of physical and cognitive impairment in China [9]. Inflammatory mechanisms have a role in the pathogenesis of ischemic stroke [10], and inflammatory cytokines have been implicated in adding to the progression of ischemic stroke [11, 12]. Galectin-3 was a microglia/macrophage-derived inflammatory mediator [13]. One study showed that the galectin-3 serum levels were associated with large artery atherosclerotic stroke [14]. A review study showed that galectin-3 was a prognostic marker and therapeutic target in cerebrovascular disease [15]. In this single-center prospective study, we included 288 consecutive patients with a first-ever acute ischemic stroke to assess the association between galectin-3 serum level and both clinical severity at admission and outcome at discharge.

## RESULTS

**Figure 1:**
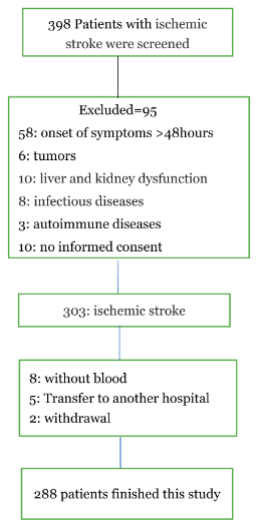
**A study flow diagram.**

As shown in Figure 1, 398 Patients with ischemic stroke were screened, and 288 patients with blood samples were analyzed at discharge (110 were excluded: 58 with the onset of symptoms >48hours, 6 with tumors, 10 with liver and kidney dysfunction, 8 with infectious diseases, 3 with autoimmune diseases, 10 with no informed consent, 8 without blood at admission, 5 Transfer to another hospital and 2 withdrawal). However, the baseline characteristics [age (P=0.18), BMI(P=0.37), sex (P=0.43) and NIHSS (P=0.09)] of those included patients were similar with the overall cohort(n=398).

As shown in Table 1, 121 (42.0%) were female, and the median age was 67 years (IQR, 59–74). The median SBP and DBP were 142(IQR, 130-175) and 90(83-95) mmHg. A total of 212 patients (73.6%) had hypertension, 48 patients (16.7%) had a positive family history of cardiovascular events, and 71 (24.7%) were diabetes mellitus. Table 1 had shown more information.

**Table 1:** Baseline characteristics of stroke patients (N=288).

**Demographic characteristics**	
Age (years), median (IQR)	67(59-74)
Male sex, n (%)	167(58.0)
BMI(Kg/m2), median (IQR)	24.8(23.1-25.9)
Clinical findings at admission, median (IQR)	
Temperature, ° C	37.0(36.5-37.3)
Systolic blood pressure, mmHg	142(130-175)
Diastolic blood pressure, mmHg	90(83-95)
Heart rate, beats/min	80(69-90)
Vascular risk factors no. (%)	
Hypertension	212(73.6)
Coronary heart disease	55(19.1)
Atrial fibrillation	29(10.0)
Hypercholesterolaemia	85(29.5)
Family history of cardiovascular event	48(16.7)
Diabetes mellitus	71(24.7)
Smoking history	56(19.4)
Drinking history	52(18.1)
Stroke aetiology no. (%)	
Large artery	65(22.6)
Small artery	52(18.1)
Cardioembolism	88(30.6)
Other cause	28(9.7)
Unknown	55(19.1)
NIHSS at admission, median (IQR)	6(4-12)
NIHSS < 6, no. (%)	132(45.8)
NIHSS >5, no. (%)	156(54.2)
Laboratory findings, median (IQR)	
Gal-3, ng/ml	7.3(5.1-11.6)
CRP, mg/l	4.5(2.0-11.2)
FSG, mmol/l	5.6(4.8-6.8)
Acute treatment, no. (%)	
IV thrombolysis	35(12.2)
Mechanical thrombectomy	10(3.5)
IV thrombolysis and/or Mechanical thrombectomy	38(13.2)
Rankin at discharge, median (IQR)	1(0-3)
mRS 0-2, no. (%)	199(69.1)
mRS 3-6, no. (%)	89(30.9)
Hospital stay, days, median (IQR)	9(5-12)
Hospital costs, Yuan, median (IQR)	23250(18145-30143)

IQR, interquartile range; NIHSS, National Institutes of Health Stroke Scale; CRP, C–reactive protein; mRS, modified Rankin Scale; FSG, fasting serum glucose; BMI, body mass index; Gal-3, galectin-3.

The median serum level of galectin-3 in those included patients was 7.3ng/ml (IQR, 5.1-11.6), with higher levels indicating increasing stroke severity. A positive relationship between NIHSS score and galectin-3 serum level had been found(r[spearman]=0.395, P<0.001). Also, the serum level of galectin-3 was related to CRP (r=0.296, P<0.001).

**Figure 2:**
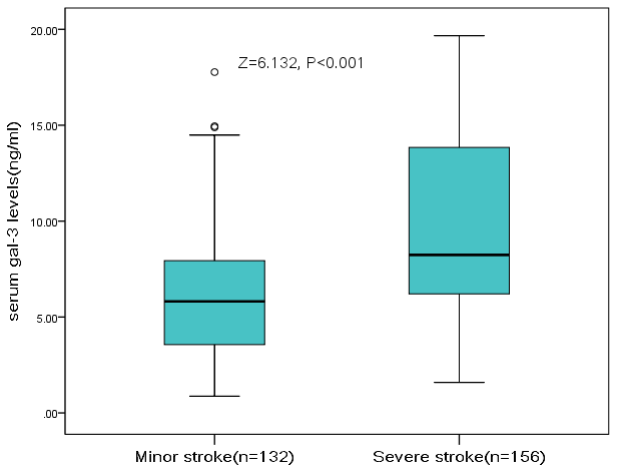
**The association between serum level of galectin-3 and stroke severity at admission.** High clinical severity was defined as a NIHSS >5, while minor stroke was defined as a NIHSS <6. Mann–Whitney U Test. All data are medians and interquartile ranges (IQR).

**Figure 3:**
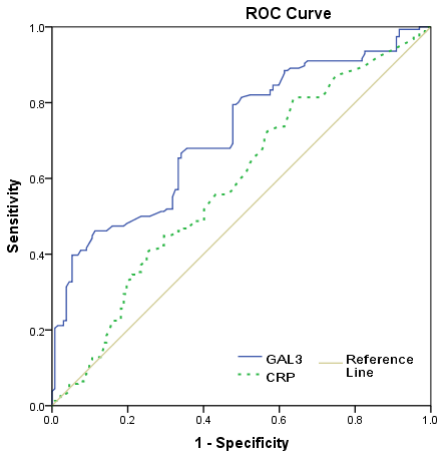
**Receiver operating characteristic (ROC) curve was utilized to evaluate the accuracy of serum level of galectin-3 to diagnose severe stroke.** High clinical severity was defined as a NIHSS >5.

The severity of the stroke was evaluated on admission. The median (IQR) NIHSS was 6 (4-12), and 132 patients (45.8%) had a minor stroke. The median (IQR) serum level of galectin-3 in patients with a moderate-to-high severity was lower than in those patients with a minor stroke (l8.2[6.2-13.8]ng/ml vs. 5.8[3.6-7.9]ng/m; P<0.001), Figure 2. According to ROC model analysis, the optimal threshold of galectin-3 to diagnose moderate-to-high stroke severity (Youden's index) was 10.0ng/ml, which shows the best prediction effect (specificity: 46.2% and sensitivity:88.6%), Figure 3. With an AUC of 0.72 (95% CI, 0.66–0.76), the diagnostic ability of galectin-3 was better than CRP (AUC, 0.58; 95% CI, 0.52–0.65; P<0.001) and age (AUC, 0.56; 95% CI, 0.51–0.63; P<0.001). Besides, CRP did not improve the diagnostic ability of galectin-3 (AUC of the combined model, 0.73; 95% CI, 0.67-0.78; P=0.62) (Table 2).

**Table 2:** Prediction of stroke severity and functional outcome.

**Parameter**	**AUC**	**95% CI**		**p**
Prediction of high clinical severity^†^				
Gal-3	0.72	0.66	0.76	<0.001
CRP	0.58	0.52	0.65	0.012
Age	0.56	0.51	0.63	0.038
Combined score (Gal-3+CRP)	0.73	0.67	0.78	<0.01
Prediction of poor outcome^‡^				
NIHSS	0.76	0.70	0.83	<0.001
Gal-3	0.76	0.70	0.82	<0.001
CRP	0.62	0.55	0.69	0.001
Age	0.60	0.53	0.66	0.010
Combined score (NIHSS+Gal-3)	0.82	0.77	0.87	<0.01

^†^High clinical severity was defined as a NIHSS >5.

^‡^Poor functional outcome was defined as a mRS 3-6.

NIHSS, National Institutes of Health Stroke Scale; CRP, C–reactive protein; Gal-3, galectin-3; AUC, area under the curve; CI= confidence interval.

Univariate and multivariate logistic analysis was used to analyze serum galectin-3 with moderate-to-high stroke severity at admission. In univariate analysis, the galectin-3 level had a strong association with high seriousness, and the risk increased 24% for one-unit(ng/ml) level increasing (OR, 1.24; 95%CI, 1.16-1.33; P<0.001). In multivariate analysis, galectin-3 remained a high stroke severity predictor with an adjusted OR of 1.16 (95% CI, 1.08–1.22; P<0.001), after adjusted for demographic characteristics, clinical findings at admission, vascular risk factors, stroke subtype, and serum levels of CRP and FSG.

**Figure 4:**
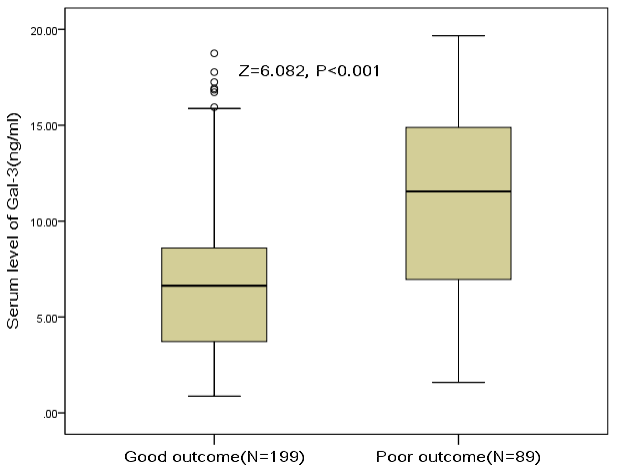
**The association between serum level of galectin-3 and stroke outcome at discharge.** Poor functional outcome was defined as an mRS 3-6, while good outcome was defined as a an mRS 0-2. Mann–Whitney U Test. All data are medians and interquartile ranges (IQR).

**Figure 5:**
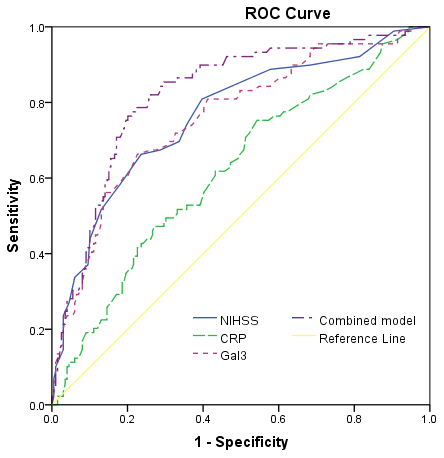
**Receiver operating characteristic (ROC) curve was utilized to evaluate the accuracy of serum level of galectin-3 to predict poor functional outcome.** Poor functional outcome was defined as an mRS 3-6.

At discharge, 89 patients had mRS scores more than 2, and the poor functional outcome rate was thus 30.9%. In these patients, the median serum level of galectin-3 was higher than in those patients with good prognosis (11.6[IQR, 6.9-14.9]ng/ml vs. 6.6[3.7-8.6]ng/ml; P<0.001), Figure 4. According to ROC model analysis, the optimal threshold of galectin-3 to predict poor functional outcome (Youden's index) was 8.6ng/ml, which shows the best prediction effect (specificity: 60.7% and sensitivity:76.4%), Figure 5. With an AUC of 0.76 (95% CI, 0.70–0.82), the discriminatory ability of galectin-3 was better than CRP (AUC, 0.62; 95% CI, 0.55–0.69; P<0.001), age (AUC, 0.60; 95% CI, 0.53–0.66; P<0.001) and was within the range of the NIHSS score (AUC, 0.76; 95% CI, 0.70–0.83; P=0.82), Table 2. Furthermore, galectin-3 could improve the predictive ability of NIHSS. The average AUC (standard error) for NIHSS and the combined model (NIHSS and galectin-3) were 0.764 (0.031) and 0.823 (0.027), corresponding to a difference of 0.059 (0.004).

Logistic regression analysis was used to analyze the association of serum galectin-3 with stroke prognosis at discharge. The multivariable model included demographic characteristics, clinical findings at admission, vascular risk factors, NIHSS at admission, stroke subtype, acute stroke treatment, and serum levels of CRP, FSG, and galectin-3. Galectin-3 level had a strong association with poor stroke prognosis and unadjusted and adjusted risk increased 25% (OR, 1.25; 95%CI, 1.17-1.33; P<0.001) and 14% (OR, 1.14; 95%CI, 1.07-1.22; P<0.001), respectively for per unit(ng/ml) level increasing. As shown in Table 3, a Galectin-3 serum level in the highest quartile (The lowest three quartiles[Q1-3] as the reference) was associated with a higher risk of poor prognosis (OR, 3.15; 95% CI, 2.44–3.87; P<0.001).

**Table 3:** Multivariate analysis of predictors of poor functional outcome.^‡^

**Factors**	**OR (95%CI)**	**P**
Gal-3 ≥11.6 ng/ml^†^	3.15(2.44-3.87)	<0.001
CRP (increase per unit)	1.05(1.02-1.09)	0.011
Age (increase per unit)	1.07(1.03-1.11)	0.002
NIHSS (increase per unit)	1.22(1.13-1.30)	<0.001
Acute treatment (Yes vs. no)	0.25(0.15-0.30)	<0.001
Stroke subtype (Large artery vs. others)	2.45(1.56-3.31)	0.040

^‡^ Multivariable model included the following variables: demographic characteristics, clinical findings at admission, vascular risk factors, NIHSS at admission, stroke subtype, acute stroke treatment and serum levels of CRP, FSG and Gal-3. Poor functional outcome was defined as an mRS 3-6.

^†^ The lowest three quartiles(Q1-3) as the reference(<11.6ng/ml).

OR, odds ratio; CI, confidence interval; NIHSS, National Institutes of Health Stroke Scale; CRP, C–reactive protein; mRS, modified Rankin Scale; FSG, fasting serum glucose; BMI, body mass index; Gal-3, galectin-3

## DISCUSSION

Finding useful blood biomarkers for predicting stroke prognosis is of great significance for the precise treatment of this disease. In this prospective study, the data shows that higher serum levels of galectin-3 are associated with stroke severity at admission and stroke prognosis at discharge in ischemic stroke. This association is independent of other factors, including age, NIHSS, and CRP. Also, as an independent prognostic marker, galectin-3 could improve the predictive efficiency of the NIHSS score.

Stroke already has some scores that predict outcomes, such as NIHSS that assesses stroke non-invasively and is also very accurate (both sensitive and specific) [16, 17]. However, in this study, we showed that galectin-3 perform better than NIH score and are additionally useful in predicting the severity and prognosis of ischemic stroke.

The prognostic value of galectin-3 for stoke patients had also been affirmed in previous studies. Wang et al. [18] reported that higher galectin-3 serum levels were related to increased risk of death or major disability in ischemic stroke during the 3-month follow-up. Zeng et al. [19] found that high serum galectin-3 levels were related to increased risk of the poor functional outcome and recurrence events in ischemic stroke patients with hyperglycemia. Yan et al. [20] found that high galectin-3 plasma levels were related to poor prognosis in acute intracerebral hemorrhage. This association also had been confirmed in patients with traumatic brain injury [21]. Furthermore, the prognostic value of plasma levels of galectin-3 in aneurysmal subarachnoid hemorrhage(aSAH) [22] and non-severe aSAH [23] had been suggested. Besides, one study showed that galectin-3 was associated with cardiovascular death in coronary artery disease patients [24].

Mostacada et al. [25] reported that the lack of galectin-3 had a role in the inflammatory process triggered by spinal cord injury (SCI) in a C57BL/6 wild-type and galectin-3 knockout mice model. A review showed that galectin-3 released by activated microglia could participate in brain immune responses [26]. Yip et al. [27] showed that the administration of neutralizing antibodies against galectin-3 exerted a neuroprotection role in the hippocampus after head injury.

Furthermore, the protective effects of galectin-3 had been proposed. One animal experiment showed a lack of galectin-3 could aggravate subchronic injury after neonatal focal stroke [28]. In a vitro study, Wesley et al. suggested that galectin-3 could play a role in post-ischemic repair [29]. Another study showed that galectin-3 could cause post-ischemic tissue remodeling by increasing angiogenesis and neurogenesis in a rat ischemic brain [30]. Furthermore, one study found that the targeted deletion of galectin-3 could aggravate ischemic damage after cerebral ischemia [31]. Rahimian et al. [32] showed that galectin-3 could induce a therapeutic shift in microglia polarization after ischemic injury. Another study showed that galectin-3 had a role in neuro-vascular protection and functional recovery after ischemic stroke, and this effect worked through modulation of angiogenic and apoptotic pathways [33]. The diverse functions might cause these conflicting findings, expression sites of galectin-3, and the time since injury [15, 26]. As an observational study, we also could not draw causal conclusions. More research needs to be performed in the future to test the relationship between the galectin-3 and stroke prognosis.

The prognostic value of galectin-3 in stroke might be involved in the Inflammation pathway. The inflammatory mechanism played a role in necrotic brain injury in stroke patients, and anti-inflammatory strategies had been suggested for treatment [34]. galectin-3 could initiate a toll-like receptor 4 (TLR4)-dependent inflammatory response in microglia, and Gal3-TLR4 interaction had been proposed [35]. Cheng et al. [36] reported that stroke could trigger major and peripheral galectin-3 release leading to enteric neuronal loss through a TLR4 mediated mechanism involving AMPK and TAK1 signaling path permanent middle cerebral artery occlusion model. Furthermore, galectin-3 was a central upstream regulator of the microglial immune response, and it could drive pro-inflammatory activation of microglia in Alzheimer's disease [37]. Galectin-3 was a prominent contributor to the pathology of atherosclerotic plaque progression by amplification of key pro-inflammatory molecules [38]. Doverhag et al. [39] found that galectin-3 caused neonatal hypoxic-ischemic brain injury through the inflammation pathway. Second, Microglia/macrophages are involved in the defense against brain damage [40]. Galectin-3 could lead to macrophage and fibroblast proliferation and fibrosis [41]. Lastly, one study found that galectin-3 was up-regulated in delayed neuronal death, and hypothermia could prevent galectin-3 up-regulation [42].

Some limitations should be acknowledged. First, we only collected serum samples at admission. We could not assess when and how long galectin-3 was changed in stroke patients. We also could not confirm the level of galectin-3 in the cerebrospinal fluid (CSF). One study found that the CSF levels of galectin-3 were increased after birth asphyxia and might contribute to injury [43]. Second, the causality conclusion could not be confirmed in this study, and whether lowering blood concentration of galectin-3 could improve the prognosis of stroke patients requires further research. A previous study showed that modified citrus pectin (a galectin-3 inhibitor) prevented mouse post-subarachnoid hemorrhage blood-brain barrier disruption, possibly inhibiting galectin-3 [44]. Third, long-term follow-up, such as 3-month and 12-month, were not included. Interestingly, the prognostic value of galectin-3 in 3-month [18] and 12-month [19] in the ischemic stroke patients had been reported. Lastly, the data derived from a single-center small sample study must be verified in future large sample multicenter studies.

## Conclusion

This study shows that higher serum levels of galectin-3 are associated with stroke severity at admission and stroke prognosis at discharge in ischemic stroke. This association was independent of other factors, including age, NIHSS, and CRP. Future studies assess pathways of lowing galectin-3 may lead to new targets for improving the prognosis of ischemic stroke.

## MATERIALS AND METHODS

From December 2017 to December 2019, all first-ever ischemic stroke patients admitted to Weifang People’s Hospital, China, were included according to the World Health Organization criteria [45] and with symptom onset within 48 hours. Disease clinical diagnosis was confirmed by computer tomography (CT), and/or magnetic resonance imaging (MRI). Patients with tumors, liver, and kidney dysfunction, infectious and autoimmune diseases, were excluded.

On admission, we use standard questionnaires to collect clinical information. That information included age, gender; vital signs; medication before stroke; systolic blood pressure (SBP) and diastolic blood pressure(DBP); smoking and drinking history; hypertension; hyperlipidemia; diabetes mellitus; atrial fibrillation; cardiovascular diseases; previous transient ischemic attack and family history of stroke. Also, acute treatment information (IV thrombolysis and/or mechanical thrombectomy) was also recorded. The severity of stroke was also assessed by a stroke neurologist (Dr. ZHOU) according to the National Institutes of Health Stroke Scale (NIHSS) score (From 0 to 42, the higher the score, the more serious of the disease) [46]. Stroke cause was classified by the original TOAST classification (Trial of Org 10172 in Acute Stroke Treatment), which including large-artery arteriosclerosis stroke; cardiac embolism; small-artery occlusion stroke; other ischemic strokes; and ischemic stroke of undetermined cause [47].

Fasting serum samples were separated and stored at -80° C until the time of analysis. A commercially available enzyme-linked immunosorbent assay (ELISAs; Human Galectin-3 Quantikine ELISA Kits, R&D Systems) was used to test galectin-3 serum levels. Serum levels of C-reactive protein (CRP) and fasting serum glucose (FSG) were also tested. At discharge, functional outcome was assessed by a stroke neurologist (Dr. ZHENG) blinded to galectin-3 levels according to the modified Rankin scale (mRS) [48]. Poor outcome representative of significant disability was defined as mRS 3-6 [49].

### Statistical analysis

The continuous variables of study patients were described by mean (standard deviation, SD) or median (interquartile range, IQR) according to whether the data were normally distributed. Data were expressed as percentages(percentage) for categorical variables. The association between galectin-3 serum level and both clinical severity at admission and outcome at discharge was assessed by univariate and multivariate logistic regression. The results were presented as odds ratios (OR) and 95% confidence intervals (CI). Serum leptin levels were categorized into quartiles, and the highest quartile(Q4) was compared to the other three quartiles (as the reference). A minor stroke at admission was defined as NIHSS <5, while moderate-to-high clinical severity was defined as NHISS>6 [50].

Each biomarker’s discriminatory value was also calculated as the area under the curve (AUC) of the receiver operating characteristic (ROC) curves. SPSS 24.0 software (SPSS Inc, Chicago, USA) was used to assess all statistical analyses, and P < 0.05 was defined as significance.

### Ethics

The Human Research Ethics Committee (HREC) of Weifang People’s Hospital checked and authorized this study plan and protocol. Written informed consent should be obtained from patients before they participate in the research.

## References

[1] Dumic J, Dabelic S, Flögel M. Galectin-3: an open-ended story. Biochim Biophys Acta. 2006; 1760:616–35. 10.1016/j.bbagen.2005.12.020 16478649

[2] Takenaka Y, Fukumori T, Raz A. Galectin-3 and metastasis. Glycoconj J. 2002; 19:543–49. 10.1023/B:GLYC.0000014084.01324.15 14758078

[3] Henderson NC, Sethi T. The regulation of inflammation by galectin-3. Immunol Rev. 2009; 230:160–71. 10.1111/j.1600-065X.2009.00794.x 19594635

[4] Chen HY, Liu FT, Yang RY. Roles of galectin-3 in immune responses. Arch Immunol Ther Exp (Warsz). 2005; 53:497–504. 16407782

[5] Hsu DK, Dowling CA, Jeng KC, Chen JT, Yang RY, Liu FT. Galectin-3 expression is induced in cirrhotic liver and hepatocellular carcinoma. Int J Cancer. 1999; 81:519–26. 10.1002/(sici)1097-0215(19990517)81:4<519::aid-ijc3>3.0.co;2-0 10225438

[6] de Boer RA, Voors AA, Muntendam P, van Gilst WH, van Veldhuisen DJ. Galectin-3: a novel mediator of heart failure development and progression. Eur J Heart Fail. 2009; 11:811–17. 10.1093/eurjhf/hfp097 19648160

[7] van Kimmenade RR, Januzzi JL Jr, Ellinor PT, Sharma UC, Bakker JA, Low AF, Martinez A, Crijns HJ, MacRae CA, Menheere PP, Pinto YM. Utility of amino-terminal pro-brain natriuretic peptide, galectin-3, and apelin for the evaluation of patients with acute heart failure. J Am Coll Cardiol. 2006; 48:1217–24. 10.1016/j.jacc.2006.03.061 16979009

[8] Sharma UC, Pokharel S, van Brakel TJ, van Berlo JH, Cleutjens JP, Schroen B, André S, Crijns HJ, Gabius HJ, Maessen J, Pinto YM. Galectin-3 marks activated macrophages in failure-prone hypertrophied hearts and contributes to cardiac dysfunction. Circulation. 2004; 110:3121–8. 10.1161/01.CIR.0000147181.65298.4D 15520318

[9] Tu WJ, Qiu HC, Zhang Y, Cao JL, Wang H, Zhao JZ, Liu Q, Zeng X. Lower serum retinoic acid level for prediction of higher risk of mortality in ischemic stroke. Neurology. 2019; 92:e1678–87. 10.1212/WNL.0000000000007261 30850446

[10] Jin R, Yang G, Li G. Inflammatory mechanisms in ischemic stroke: role of inflammatory cells. J Leukoc Biol. 2010; 87:779–89. 10.1189/jlb.1109766 20130219 PMC2858674

[11] Tuttolomondo A, Di Raimondo D, di Sciacca R, Pinto A, Licata G. Inflammatory cytokines in acute ischemic stroke. Curr Pharm Des. 2008; 14:3574–89. 10.2174/138161208786848739 19075734

[12] Basic Kes V, Simundic AM, Nikolac N, Topic E, Demarin V. Pro-inflammatory and anti-inflammatory cytokines in acute ischemic stroke and their relation to early neurological deficit and stroke outcome. Clin Biochem. 2008; 41:1330–4. 10.1016/j.clinbiochem.2008.08.080 18801351

[13] Dragomir AC, Sun R, Mishin V, Hall LB, Laskin JD, Laskin DL. Role of galectin-3 in acetaminophen-induced hepatotoxicity and inflammatory mediator production. Toxicol Sci. 2012; 127:609–19. 10.1093/toxsci/kfs117 22461450 PMC3355315

[14] He XW, Li WL, Li C, Liu P, Shen YG, Zhu M, Jin XP. Serum levels of galectin-1, galectin-3, and galectin-9 are associated with large artery atherosclerotic stroke. Sci Rep. 2017; 7:40994. 10.1038/srep40994 28112232 PMC5256273

[15] Venkatraman A, Hardas S, Patel N, Singh Bajaj N, Arora G, Arora P. Galectin-3: an emerging biomarker in stroke and cerebrovascular diseases. Eur J Neurol. 2018; 25:238–46. 10.1111/ene.13496 29053903

[16] Katan M, Fluri F, Morgenthaler NG, Schuetz P, Zweifel C, Bingisser R, Müller K, Meckel S, Gass A, Kappos L, Steck AJ, Engelter ST, Müller B, Christ-Crain M. Copeptin: a novel, independent prognostic marker in patients with ischemic stroke. Ann Neurol. 2009; 66:799–808. 10.1002/ana.21783 20035506

[17] Adams HP Jr, Davis PH, Leira EC, Chang KC, Bendixen BH, Clarke WR, Woolson RF, Hansen MD. Baseline NIH Stroke Scale score strongly predicts outcome after stroke: A report of the Trial of Org 10172 in Acute Stroke Treatment (TOAST). Neurology. 1999; 53:126–31. 10.1212/wnl.53.1.126 10408548

[18] Wang A, Zhong C, Zhu Z, Xu T, Peng Y, Xu T, Peng H, Chen CS, Wang J, Ju Z, Li Q, Geng D, Sun Y, et al. Serum Galectin-3 and Poor Outcomes Among Patients With Acute Ischemic Stroke. Stroke. 2018; 49:211–214. 10.1161/STROKEAHA.117.019084 29229724

[19] Zeng N, Wang A, Zhong C, Zheng X, Zhu Z, Xu T, Peng Y, Peng H, Li Q, Ju Z, Geng D, Zhang Y, He J. Association of serum galectin-3 with risks of death and vascular events in acute ischaemic stroke patients: the role of hyperglycemia. Eur J Neurol. 2019; 26:415–21. 10.1111/ene.13856 30414289

[20] Yan XJ, Yu GF, Jie YQ, Fan XF, Huang Q, Dai WM. Role of galectin-3 in plasma as a predictive biomarker of outcome after acute intracerebral hemorrhage. J Neurol Sci. 2016; 368:121–27. 10.1016/j.jns.2016.06.071 27538613

[21] Shen YF, Yu WH, Dong XQ, Du Q, Yang DB, Wu GQ, Zhang ZY, Wang H, Jiang L. The change of plasma galectin-3 concentrations after traumatic brain injury. Clin Chim Acta. 2016; 456:75–80. 10.1016/j.cca.2016.02.029 26944570

[22] Liu H, Liu Y, Zhao J, Liu H, He S. Prognostic value of plasma galectin-3 levels after aneurysmal subarachnoid hemorrhage. Brain Behav. 2016; 6:e00543. 10.1002/brb3.543 27781149 PMC5064347

[23] Nishikawa H, Nakatsuka Y, Shiba M, Kawakita F, Fujimoto M, Suzuki H, and pSEED Group. Increased Plasma Galectin-3 Preceding the Development of Delayed Cerebral Infarction and Eventual Poor Outcome in Non-Severe Aneurysmal Subarachnoid Hemorrhage. Transl Stroke Res. 2018; 9:110–119. 10.1007/s12975-017-0564-0 28831694

[24] Maiolino G, Rossitto G, Pedon L, Cesari M, Frigo AC, Azzolini M, Plebani M, Rossi GP. Galectin-3 predicts long-term cardiovascular death in high-risk patients with coronary artery disease. Arterioscler Thromb Vasc Biol. 2015; 35:725–32. 10.1161/ATVBAHA.114.304964 25614283

[25] Mostacada K, Oliveira FL, Villa-Verde DM, Martinez AM. Lack of galectin-3 improves the functional outcome and tissue sparing by modulating inflammatory response after a compressive spinal cord injury. Exp Neurol. 2015; 271:390–400. 10.1016/j.expneurol.2015.07.006 26183316

[26] Nishikawa H, Suzuki H. Possible role of inflammation and galectin-3 in brain injury after subarachnoid hemorrhage. Brain Sci. 2018; 8:30. 10.3390/brainsci8020030 29414883 PMC5836049

[27] Yip PK, Carrillo-Jimenez A, King P, Vilalta A, Nomura K, Chau CC, Egerton AM, Liu ZH, Shetty AJ, Tremoleda JL, Davies M, Deierborg T, Priestley JV, et al. Galectin-3 released in response to traumatic brain injury acts as an alarmin orchestrating brain immune response and promoting neurodegeneration. Sci Rep. 2017; 7:41689. 10.1038/srep41689 28128358 PMC5269662

[28] Chip S, Fernández-López D, Li F, Faustino J, Derugin N, Vexler ZS. Genetic deletion of galectin-3 enhances neuroinflammation, affects microglial activation and contributes to sub-chronic injury in experimental neonatal focal stroke. Brain Behav Immun. 2017; 60:270–81. 10.1016/j.bbi.2016.11.005 27836669 PMC7909718

[29] Wesley UV, Vemuganti R, Ayvaci ER, Dempsey RJ. Galectin-3 enhances angiogenic and migratory potential of microglial cells via modulation of integrin linked kinase signaling. Brain Res. 2013; 1496:1–9. 10.1016/j.brainres.2012.12.008 23246924 PMC4084961

[30] Yan YP, Lang BT, Vemuganti R, Dempsey RJ. Galectin-3 mediates post-ischemic tissue remodeling. Brain Res. 2009; 1288:116–24. 10.1016/j.brainres.2009.06.073 19573520

[31] Lalancette-Hébert M, Swarup V, Beaulieu JM, Bohacek I, Abdelhamid E, Weng YC, Sato S, Kriz J. Galectin-3 is required for resident microglia activation and proliferation in response to ischemic injury. J Neurosci. 2012; 32:10383–95. 10.1523/JNEUROSCI.1498-12.2012 22836271 PMC6703730

[32] Rahimian R, Lively S, Abdelhamid E, Lalancette-Hebert M, Schlichter L, Sato S, Kriz J. Delayed galectin-3-mediated reprogramming of microglia after stroke is protective. Mol Neurobiol. 2019; 56:6371–85. 10.1007/s12035-019-1527-0 30798442

[33] Wesley UV, Sutton IC, Cunningham K, Jaeger JW, Phan AQ, Hatcher JF, Dempsey RJ. Galectin-3 protects against ischemic stroke by promoting neuro-angiogenesis via apoptosis inhibition and Akt/Caspase regulation. J Cereb Blood Flow Metab. 2020. [Epub ahead of print]. 10.1177/0271678X20931137 PMC7983501 33736511

[34] Wang Q, Tang XN, Yenari MA. The inflammatory response in stroke. J Neuroimmunol. 2007; 184:53–68. 10.1016/j.jneuroim.2006.11.014 17188755 PMC1868538

[35] Burguillos MA, Svensson M, Schulte T, Boza-Serrano A, Garcia-Quintanilla A, Kavanagh E, Santiago M, Viceconte N, Oliva-Martin MJ, Osman AM, Salomonsson E, Amar L, Persson A, et al. Microglia-secreted galectin-3 acts as a toll-like receptor 4 ligand and contributes to microglial activation. Cell Rep. 2015; 10:1626–38. 10.1016/j.celrep.2015.02.012 25753426

[36] Cheng X, Boza-Serrano A, Turesson MF, Deierborg T, Ekblad E, Voss U. Galectin-3 causes enteric neuronal loss in mice after left sided permanent middle cerebral artery occlusion, a model of stroke. Sci Rep. 2016; 6:32893. 10.1038/srep32893 27612206 PMC5017186

[37] Boza-Serrano A, Ruiz R, Sanchez-Varo R, García-Revilla J, Yang Y, Jimenez-Ferrer I, Paulus A, Wennström M, Vilalta A, Allendorf D, Davila JC, Stegmayr J, Jiménez S, et al. Galectin-3, a novel endogenous TREM2 ligand, detrimentally regulates inflammatory response in Alzheimer’s disease. Acta Neuropathol. 2019; 138:251–73. 10.1007/s00401-019-02013-z 31006066 PMC6660511

[38] Papaspyridonos M, McNeill E, de Bono JP, Smith A, Burnand KG, Channon KM, Greaves DR. Galectin-3 is an amplifier of inflammation in atherosclerotic plaque progression through macrophage activation and monocyte chemoattraction. Arterioscler Thromb Vasc Biol. 2008; 28:433–40. 10.1161/ATVBAHA.107.159160 18096829

[39] Doverhag C, Hedtjärn M, Poirier F, Mallard C, Hagberg H, Karlsson A, Sävman K. Galectin-3 contributes to neonatal hypoxic-ischemic brain injury. Neurobiol Dis. 2010; 38:36–46. 10.1016/j.nbd.2009.12.024 20053377

[40] Xiong XY, Liu L, Yang QW. Functions and mechanisms of microglia/macrophages in neuroinflammation and neurogenesis after stroke. Prog Neurobiol. 2016; 142:23–44. 10.1016/j.pneurobio.2016.05.001 27166859

[41] Aguilar D, Sun C, Hoogeveen RC, Nambi V, Selvin E, Matsushita K, Saeed A, McEvoy JW, Shah AM, Solomon SD, Boerwinkle E, Ballantyne CM. Levels and change in galectin-3 and association with cardiovascular events: the ARIC study. J Am Heart Assoc. 2020; 9:e015405. 10.1161/JAHA.119.015405 32573308 PMC7670497

[42] Satoh K, Niwa M, Goda W, Binh NH, Nakashima M, Takamatsu M, Hara A. Galectin-3 expression in delayed neuronal death of hippocampal CA1 following transient forebrain ischemia, and its inhibition by hypothermia. Brain Res. 2011; 1382:266–74. 10.1016/j.brainres.2011.01.049 21262205

[43] Sävman K, Heyes MP, Svedin P, Karlsson A. Microglia/macrophage-derived inflammatory mediators galectin-3 and quinolinic acid are elevated in cerebrospinal fluid from newborn infants after birth asphyxia. Transl Stroke Res. 2013; 4:228–35. 10.1007/s12975-012-0216-3 23807898 PMC3685715

[44] Nishikawa H, Liu L, Nakano F, Kawakita F, Kanamaru H, Nakatsuka Y, Okada T, Suzuki H. Modified Citrus Pectin Prevents Blood-Brain Barrier Disruption in Mouse Subarachnoid Hemorrhage by Inhibiting Galectin-3. Stroke. 2018; 49:2743–2751. 10.1161/STROKEAHA.118.021757 30355205

[45] Hatano S. Experience from a multicentre stroke register: a preliminary report. Bull World Health Organ. 1976; 54:541–53. 1088404 PMC2366492

[46] Brott T, Marler JR, Olinger CP, Adams HP Jr, Tomsick T, Barsan WG, Biller J, Eberle R, Hertzberg V, Walker M. Measurements of acute cerebral infarction: lesion size by computed tomography. Stroke. 1989; 20:871–75. 10.1161/01.str.20.7.871 2749847

[47] Adams HP Jr, Bendixen BH, Kappelle LJ, Biller J, Love BB, Gordon DL, Marsh EE 3rd. Classification of subtype of acute ischemic stroke. Definitions for use in a multicenter clinical trial. TOAST. Trial of ORG 10172 in acute stroke treatment. Stroke. 1993; 24:35–41. 10.1161/01.str.24.1.35 7678184

[48] Bonita R, Beaglehole R. Recovery of motor function after stroke. Stroke. 1988; 19:1497–500. 10.1161/01.str.19.12.1497 3201508

[49] Tu WJ, Dong X, Zhao SJ, Yang DG, Chen H. Prognostic value of plasma neuroendocrine biomarkers in patients with acute ischaemic stroke. J Neuroendocrinol. 2013; 25:771–78. 10.1111/jne.12052 23701638

[50] Daubail B, Jacquin A, Guilland JC, Hervieu M, Osseby GV, Rouaud O, Giroud M, Béjot Y. Serum 25-hydroxyvitamin D predicts severity and prognosis in stroke patients. Eur J Neurol. 2013; 20:57–61. 10.1111/j.1468-1331.2012.03758.x 22632854

